# Reassessing Pulmonary Hypertension Classification: Utilizing Criteria for Heart Failure with Preserved Ejection Fraction Instead of Pulmonary Arterial Wedge Pressure

**DOI:** 10.3390/jcm13247582

**Published:** 2024-12-13

**Authors:** Da-Hee Park, Jan Fuge, Jan Christopher Kamp, Britta Harrigfeld, Dominik Berliner, Marius M. Hoeper, Karen M. Olsson

**Affiliations:** 1 Department of Respiratory Medicine and Infectious Diseases, Hannover Medical School, Carl-Neuberg-Str. 1, 30625 Hannover, Germany; 2 Biomedical Research in Endstage and Obstructive Lung Disease Hannover (BREATH), German Center for Lung Research (DZL), 30625 Hannover, Germany; 3 Department of Cardiology and Angiology, Hannover Medical School, Carl-Neuberg-Str. 1, 30625 Hannover, Germany

**Keywords:** pulmonary hypertension, idiopathic pulmonary arterial hypertension, left heart disease, heart failure with preserved ejection fraction, pulmonary arterial wedge pressure

## Abstract

**Background**: The current classification of pulmonary hypertension (PH) distinguishes between pre-capillary (PAWP ≤ 15 mmHg) and post-capillary (PAWP > 15 mmHg) forms, with left heart disease, especially heart failure with preserved ejection fraction (HFpEF), being a common cause of PH. We investigated the suitability of an HFpEF diagnosis instead of PAWP in diagnosing PH associated with HFpEF. **Methods**: In a retrospective, single-center analysis, we reviewed diagnoses from our database, focusing on patients initially diagnosed with idiopathic pulmonary arterial hypertension (IPAH) or PH associated with HFpEF (PH-HFpEF) based on their PAWP. These patients were reclassified, distinguishing between HFpEF and non-HFpEF cases. Patients with PH-HFpEF were further stratified by PAWP (≤15 mmHg or >15 mmHg). **Results**: The study included 350 patients: 214 (61.1%) with PAWP ≤ 15 mmHg and 136 (38.9%) with PAWP > 15 mmHg. Replacing the PAWP criterion with the HFpEF criterion resulted in the reclassification of 121 of 350 (34.6%) patients (115 of 214 [53.7%] from IPAH to PH-HFpEF and 6 of 136 [4.4%] from PH-HFpEF to IPAH). The final disposition was IPAH (n = 105, 30%), PH-HFpEF with PAWP ≤ 15 mmHg (n = 115, 32.9%), and PH-HFpEF with PAWP > 15 mmHg (n = 130, 37.1%). Characteristics such as age distribution, functional impairment, co-morbidities, echocardiographic indices of HFpEF, pulmonary vascular resistance, response to PH medications, and unadjusted survival were comparable between the two HFpEF cohorts but differed substantially from those with IPAH. **Conclusions**: PH-HFpEF patients with PAWP ≤ 15 mmHg resemble those with PAWP > 15 mmHg but differ from IPAH cases. Incorporating non-invasive HFpEF criteria could refine PH diagnostic classification.

## 1. Introduction

Pulmonary hypertension (PH), characterized by a mean pulmonary arterial pressure (mPAP) above 20 mmHg (formerly ≥25 mmHg), is a prevalent hemodynamic condition affecting roughly 1% of adults worldwide [1,2,3]. It is classified both hemodynamically, based on pulmonary arterial wedge pressure (PAWP), and clinically into five groups: pulmonary arterial hypertension (PAH, group 1), PH associated with left heart disease (group 2), PH associated with lung disease (group 3), PH associated with pulmonary artery obstructions (group 4), and PH with unclear or multifactorial mechanisms (group 5). Diagnosis of group 2 PH requires a PAWP (or left ventricular end-diastolic pressure, LVEDP) ≥ 15 mmHg, while other groups typically present with pre-capillary PH, particularly idiopathic PAH (IPAH).

However, diagnosing PH can be complex due to overlapping etiologies. IPAH, historically associated with younger individuals [4], is now more prevalent in older patients with comorbidities such as hypertension, obesity, diabetes mellitus, and coronary artery disease [5,6,7,8]. These comorbidities, coupled with advanced age, heighten the risk of left heart disease, particularly heart failure with preserved ejection fraction (HFpEF), a common cause of PH [9,10]. Patients with pre-capillary PH and no identifiable causes are often labeled as IPAH with comorbidities, yet they likely manifest HFpEF. Relying solely on PAWP criteria (at rest) may obscure the diagnosis of group 2 PH in patients with PAWP ≤ 15 mmHg, potentially leading to inappropriate treatment decisions.

We hypothesized that in patients with PH and risk factors for HFpEF, rather than relying on PAWP, it may be more appropriate to diagnose HFpEF using established criteria, and to classify such patients as group 2 PH once the HFpEF diagnosis is confirmed, irrespective of their PAWP.

## 2. Methods

### 2.1. Database

The PH program at Hannover Medical School, Hannover, Germany, utilizes a data base capturing all medically relevant information of patients referred for evaluation and the management of patients with PH. Patient informed consent was waived because of the retrospective, non-interventional nature of this study. The authors had access to information that could identify individual participants during data collection. Ethical approval was obtained from the institutional review board (11302_BO_K_2024).

### 2.2. Patient Selection

Patients were selected by the following criteria: adult patients newly diagnosed with PH (defined by an mPAP ≥ 25 mmHg, measured during right heart catheterization) between 1 January 2015 and 31 October 2023; clinical diagnosis of IPAH or PH-HFpEF; echocardiography available to determine HFpEF criteria [11]; initiation of medications approved for PAH; and at least one follow-up assessment after treatment initiation. Exclusion criteria were other PH/PAH subtypes, left ventricular ejection fraction (LVEF) < 50%, and significant valvular heart disease, i.e., more than mild mitral or aortic valve stenosis or more than moderate mitral or aortic regurgitation. The final data extraction for the present analysis was performed on 16 March 2024.

### 2.3. Right Heart Catheterization

Right heart catheterization was performed in accordance with published guidelines [1,12]. The pressure transducers were zeroed in the mid thoracic line, i.e., the level of the left atrium [13]. PAWP measurements were taken at end-expiration during uninterrupted respiratory cycles. Cardiac output was determined by thermodilution.

### 2.4. Classification Criteria

The diagnosis of HFpEF relied on a modified algorithm proposed by the *Heart Failure Association* of the *European Society of Cardiology* (HFA-PEFF) [11]. According to this algorithm, HFpEF is considered in patients exhibiting symptoms or signs of heart failure, a normal LVEF, the absence of significant heart valve disease or cardiac ischemia, and the presence of at least one typical risk factor (obesity, hypertension, diabetes mellitus, elderly, atrial fibrillation). In individuals meeting these criteria, the confirmation of HFpEF diagnosis involves echocardiography and natriuretic peptides. The echocardiographic measures include mitral annular early diastolic velocity (e′), left ventricular (LV) filling pressure estimated using E/e′, left atrial volume index (LAVI), LV mass index (LVMI), LV relative wall thickness, tricuspid regurgitation velocity, and LV global longitudinal systolic strain.

For our analysis, we excluded the biomarker criterion, as the presence of PH led to elevated NT-proBNP levels in most patients, regardless of the presence or absence of HFpEF. Similarly, we excluded tricuspid regurgitation velocity, a marker of PH rather than HFpEF. Global longitudinal strain was omitted since it was not routinely captured during the timeframe of this analysis. Table 1 displays a comparison between the original HFA-PEFF criteria and the modified criteria used in this study. For this study, a HFpEF diagnosis was considered established when at least one clinical risk factor along with at least one major and one minor echocardiographic criterion of HFpEF were present.

A secondary analysis was conducted using PAWP ≤ 12 versus >12 mmHg as the starting point, followed by the same analyses as for the PAWP ≤ 15 versus >15 mmHg cut-off level.

### 2.5. Statistical Analyses

Statistical analyses were conducted using SPSS version 29 (IBM, Armond, NY, USA) and R software, major version 4 (The R consortium, available online via www.r-project.org). Categorical data are presented as numbers and percentages, while continuous data are expressed as the median along with the first and third quartiles [Q1, Q3]. The initial follow-up was defined as the first assessment within 3 to 12 months after the initiation of treatment. Vital status was determined through on-site visits or phone calls to the patients, their relatives, or their primary care physicians. Patients who underwent lung transplantation were censored at the transplantation date, and those lost to follow-up were censored at the date of the last contact.

To compare patient cohorts with IPAH and the two cohorts with PH-HFpEF, two-sample t-tests or Wilcoxon rank sum tests were employed for continuous data. Categorical data were compared using Pearson’s Chi-squared test or Fisher’s exact test.

Response to therapy was assessed by changes from baseline to the first follow-up in WHO functional class (FC), 6 min walking distance (6MWD), N-terminal fragment of pro-brain natriuretic peptide (NT-proBNP), and mortality risk, using the ESC/ERS 4-strata model [14]. These analyses were descriptive, i.e., without statistical comparisons. Survival estimates from the time of PH diagnosis were determined through Kaplan–Meier analyses and log-rank tests. Cox proportional hazard regression analysis was conducted to adjust for age. *p*-values < 0.05 were considered statistically significant.

## 3. Results

### 3.1. Patients

**Figure 1:**
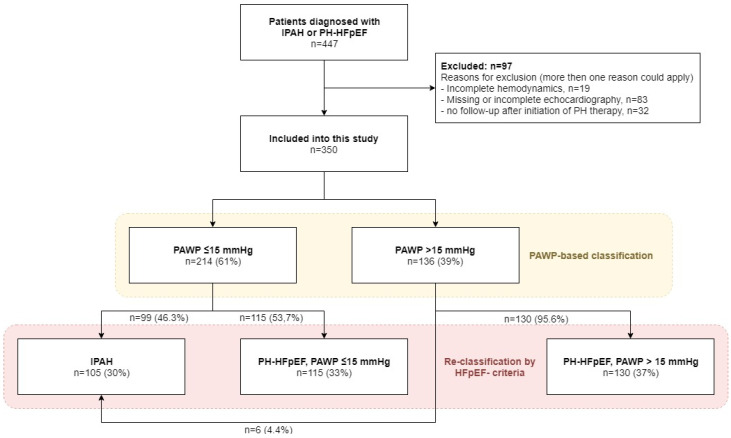
Scope diagram showing patient selection. Definition of abbreviations: IPAH, idiopathic pulmonary arterial hypertension; PH, pulmonary hypertension; HFpEF, heart failure with preserved ejection fraction; PAWP, pulmonary arterial wedge pressure.

Patient selection and reasons for exclusion are shown in Figure 1. After the exclusion of patients who did not fulfil the selection criteria, this study included a total of 350 patients: 214 (61.1%) with PAWP ≤ 15 mmHg and 136 (38.9%) with PAWP > 15 mmHg. Patients’ demographics and characteristics based on PAWP ≤ 15 mmHg vs. >15 mmHg are shown in Supplementary Table S1. The age distribution of these two groups is shown in Supplementary Figure S1. A sizable proportion of patients with PH-HFpEF presented with atrial fibrillation. A comparison of patients with and without atrial fibrillation is shown in Supplementary Table S3.

When the PAWP criterion was replaced by the HFpEF criterion, 121 of 350 (34.6%) were reclassified. Of 214 patients with PAWP ≤ 15 mmHg, 99 (46.3%) had no signs of HFpEF and were classified as IPAH, while the remaining 115 (53.7%) patients fulfilled the HFpEF criteria and were reclassified as PH-HFpEF with PAWP ≤ 15 mmHg. Among the 136 patients with PAWP > 15 mmHg, 6 (4.4%) had no signs of HFpEF and were reclassified as IPAH (the clinical data of these patients are shown in Supplementary Table S2). The remaining 130 (95.6%) patients with PAWP > 15 mmHg fulfilled the HFpEF criteria and were categorized as PH-HFpEF with PAWP > 15 mmHg.

**Figure 2:**
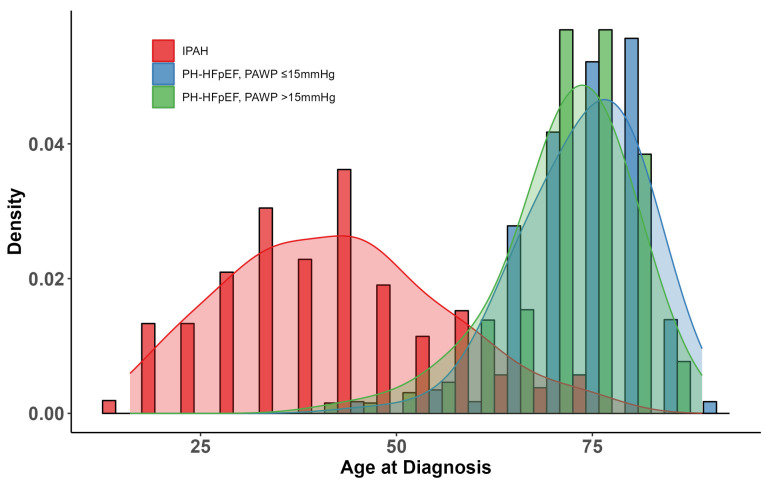
Age distribution of patients classified as IPAH, PH-HFpEF with PAWP ≤ 15 mmHg, and PH-HFpEF with PAWP > 15 mmHg. Density plots showing the age distribution of patients with IPAH (red), PH-HFpEF with PAWP ≤ 15 mmHg (blue), and PH-HFpEF with PAWP > 15 mmHg (green). Definition of abbreviations: IPAH, idiopathic pulmonary arterial hypertension; PH, pulmonary hypertension; HFpEF, heart failure with preserved ejection fraction; PAWP, pulmonary arterial wedge pressure.

Hence, the final patient disposition was IPAH (n = 105, 30%), PH-HFpEF with PAWP ≤ 15 mmHg (n = 115, 32.9%), and PH-HFpEF with PAWP > 15 mmHg (n = 130, 37.1%). Patient characteristics at the time of PH diagnosis are shown in Table 2. The age distributions of the two HFpEF cohorts were comparable, as shown in Figure 2. Compared to patients with PH-HFpEF, patients with IPAH were about three decades younger and less functionally impaired despite having higher mPAP, higher pulmonary vascular resistance (PVR), and lower cardiac index (CI). In contrast, the two PH-HFpEF cohorts had comparable baseline characteristics. Both PH-HFpEF cohorts had a similar comorbidity profile, driven by a high prevalence of hypertension and diabetes. A history of atrial fibrillation was present in 66% and 64% of the patients in the two HFpEF cohorts. The hemodynamic profile of patients with PH-HFpEF with PAWP ≤ 15 mmHg and >15 mmHg was remarkable, as both populations had comparable values of PVR, CI, and mixed-venous oxygen saturation SvO_2_, while right atrial pressure, mPAP, and PAWP were all significantly higher in the PH-HFpEF with PAWP > 15 mmHg cohort. Of note, the median [Q1, Q3] PAWP in patients with PH-HFpEF and PAWP ≤ 15 mmHg was 11 [9, 14] mmHg.

Echocardiography showed substantial differences between patients with IPAH and patients with HFpEF and PAWP ≤ 15 mmHg in E/e′, LAVI, and LVMI, while E/e′ and LAVI were comparable in the two PH-HFpEF populations, with less pronounced left ventricular hypertrophy in patients with PH-HFpEF and PAWP ≤ 15 mmHg than in those with PAWP > 15 mmHg (Table 3).

The echocardiographic parameters of the two cohorts grouped by PAWP ≤ 15 mmHg versus >15 mmHg are shown in Supplementary Table S4.

### 3.2. Changes in WHO-FC, 6MWD, NT-proBNP, and Risk After Initiation of PH Therapy

Per inclusion criteria, all patients received medications approved for PAH, but treatment patterns varied substantially. Combination therapy was used in most patients with IPAH, while monotherapy—mostly with phosphodiesterase-5 inhibitors—was the predominant treatment choice in the two PH-HFpEF cohorts.

**Figure 3:**
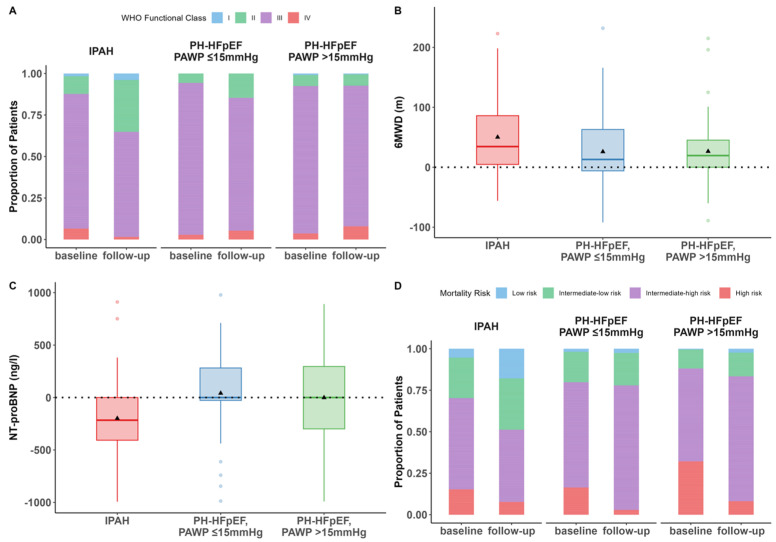
Baseline and first follow-up after initiation of therapy for (**A**) WHO functional class, (**B**) 6 min walk distance, (**C**) N-terminal fragment of pro-brain natriuretic peptide, and (**D**) mortality risk as determined by the ESC/ERS 4-strata model in patients classified as IPAH, PH-HFpEF with PAWP ≤ 15 mmHg, and PH-HFpEF with PAWP > 15 mmHg. (**A**): Bar graphs of WHO functional class at baseline and first follow-up after treatment initiation. (**B**) Box and whisker plots showing changes from baseline to first follow-up in 6 min walk distance. (**C**) Box and whisker plots showing changes from baseline to first follow-up in NT-proBNP. (**D**): Bar graphs showing distribution of mortality risk estimated by the ESC/ERS 4-strata model at baseline and first follow-up. The triangles in (**B**,**C**) represent the respective mean changes. Definition of abbreviations: IPAH, idiopathic pulmonary arterial hypertension; PH, pulmonary hypertension; HFpEF, heart failure with preserved ejection fraction; PAWP, pulmonary arterial wedge pressure.

The median [Q1, Q3] interval between baseline and first follow-up after initiation of PH therapy was 4 months [3, 6]. WHO-FC at baseline and first follow-up is shown in Figure 3A. In patients with IPAH, WHO-FC improved, did not change, or deteriorated in 35 of 105 (33%), 70 of 105 (67%), and 0 of 105 (0%) patients, respectively. In patients with PH-HFpEF and PAWP ≤ 15 mmHg, the corresponding numbers were 14 of 106 (13%), 87 of 106 (82%), and 5 of 106 (5%). In patients with PH-HFpEF and PAWP > 15 mmHg, the respective numbers were 7 of 121 (16%), 106 of 121 (88%), and 13 of 121 (4%).

Median changes in 6MWD in patients with IPAH, PH-HFpEF with PAWP ≤ 15 mmHg, and PH-HFpEF with PAWP > 15 mmHg were 41 [6, 100] m, 13 [−10, 64] m, and 19 [0, 46] m (Figure 3B).

Median changes in NT-proBNP in patients with IPAH, PH-HFpEF with PAWP ≤ 15 mmHg, and PH-HFpEF with PAWP > 15 mmHg were −381 [−1226, −5] ng/L, 0 [−438, 295] ng/L, and −11 [−794 to 402] ng/L, respectively (Figure 3C).

The estimated mortality risk per the ESC/ERS 4-strata model at baseline and first follow-up is shown in Figure 3D. In patients with IPAH, risk improved, did not change, or deteriorated in 52 of 102 (51%), 41 of 102 (40%), and 9 of 102 (9%) patients, respectively. In patients with PH-HFpEF and PAWP ≤ 15 mmHg, the corresponding numbers were 23 of 105 (22%), 68 of 105 (65%), and 14 of 105 (13%). In patients with PH-HFpEF and PAWP > 15 mmHg, the respective numbers were 29 of 106 (27%), 67 of 106 (63%), and 10 of 106 (9%).

Treatment response in the two patient cohorts grouped by PAWP ≤ 15 mmHg versus >15 mmHg is shown in Supplementary Figure S2A–D.

### 3.3. Survival

**Figure 4:**
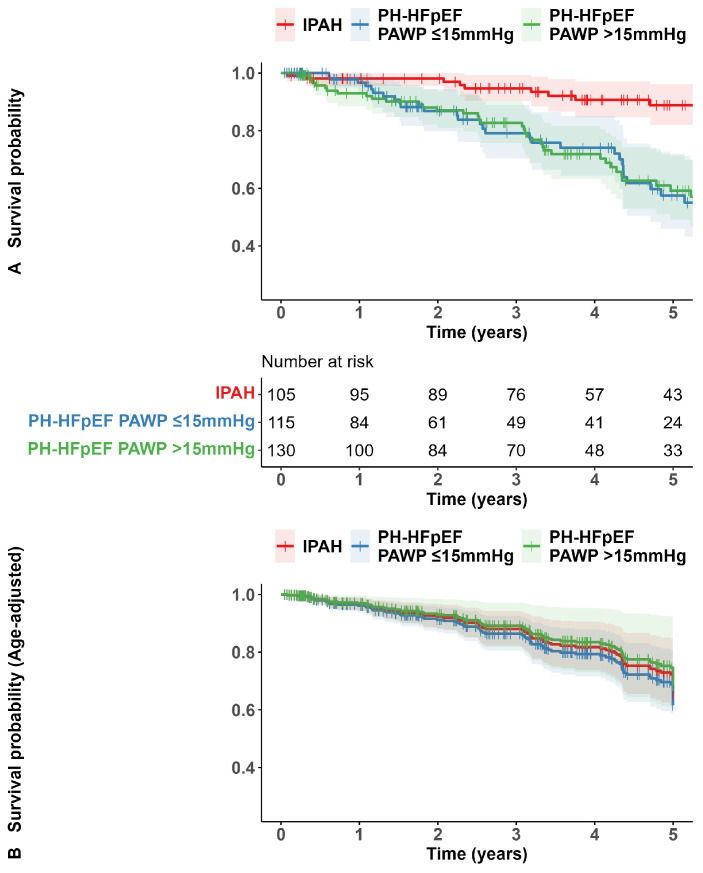
Kaplan–Meier survival estimates (**A**) and age-adjusted survival estimates (**B**) for patients classified as IPAH, PH-HFpEF with PAWP ≤ 15 mmHg, and PH-HFpEF with PAWP > 15 mmHg. Definition of abbreviations: IPAH, idiopathic pulmonary arterial hypertension; PH, pulmonary hypertension; HFpEF, heart failure with preserved ejection fraction; PAWP, pulmonary arterial wedge pressure.

The median observation time was 4.4 [2.7, 6.8] years in patients with IPAH, 2.2 [0.9, 4.7] years in patients with PH-HFpEF with PAWP ≤ 15 mmHg, and 3.2 [1.1, 5.1] years in patients with PH-HFpEF and PAWP > 15 mmHg. Three (3%), 13 (11%), and 13 (10%) patients were lost to follow-up. In the IPAH cohort, 10 of 105 (9.5%) patients died, and 5 (4.8%) underwent lung transplantation (these patients were censored at the time of transplantation). In this cohort, the Kaplan–Meier estimated survival rates at 1, 3, and 5 years were 98.1%, 94.6%, and 88.1%, respectively. In the two PH-HFpEF cohorts, no transplantations occurred. There were 27 deaths of the 115 (23.5%) patients with PAWP ≤ 15 mmHg and 35 deaths of the 130 (26.9%) patients with PAWP > 15 mmHg. In these cohorts, the Kaplan–Meier estimated survival rates at 1, 3, and 5 years were 96.7%. 79.1%, and 57.5%, and 93%, 82.7%, and 59.2%, respectively (Figure 4A). The unadjusted survival differences between the IPAH cohort and the two PH-HFpEF cohorts were statistically significant (*p* < 0.001), while the survival estimates of the two PH-HFpEF populations were nearly identical (*p* = 0.968). However, when adjusted for age, the survival differences between the IPAH cohort and the two PH-HFpEF cohorts were no longer statistically significant (*p* = 0.834 and *p* = 0.613, respectively, Figure 4B).

Kaplan–Meier survival estimates of patients and age-adjusted survival estimates grouped by PAWP ≤ 15 mmHg versus >15 mmHg are shown in Supplementary Figure S3.

### 3.4. Secondary Analysis Based on PAWP ≤ 12 Versus >12 mmHg

Baseline characteristics and echocardiographic parameters of patients grouped by PAWP ≤ 12 versus 12 mmHg are shown in Supplementary Tables S5 and S6, respectively. When applying the HFpEF criteria to these cohorts, 68 of 159 (42.8%) patients with PAWP ≤ 12 mmHg were reclassified as PH-HFpEF, while 14 of 191 (7.3%) patients with PAWP > 12 mmHg were reclassified as IPAH (Figure S4). Between-group discrimination by age (Figure S5) and treatment response (Figure S6) was comparable to the PAWP ≤ 15 versus ≥15 mmHg analyses, while the survival difference between patients with PAWP ≤ 12 mmHg and >12 mmHg was not statistically significant (Figure S7).

## 4. Discussion

Presently, PH is categorized into pre-capillary and post-capillary forms based on a PAWP threshold of ≤15 mmHg or >15 mmHg, respectively [1]. The diagnosis of PH associated with left heart disease mandates a PAWP > 15 mmHg. However, patients with PH-HFpEF may manifest a PAWP ≤ 15 mmHg. In our study, utilizing HFpEF criteria instead of PAWP for PH classification resulted in more than half of the patients with PAWP ≤ 15 mmHg being re-classified from IPAH to PH-HFpEF. These patients exhibited disease characteristics like those with PAWP > 15 mmHg but differed from those with IPAH. In addition, occasional patients with well-characterized IPAH whose PAWP exceeded 15 mmHg without signs of left heart disease were accurately classified when the PAWP criterion was excluded.

Since the first PH World Symposium in 1973 [15], PAWP (or LVEDP, if PAWP cannot be reliably measured) has been used to distinguish between pre-capillary and post-capillary PH. While there has been ongoing debate about the optimal cut-off value, the utility of PAWP itself has not been seriously questioned, which is surprising, as PAWP can be affected by numerous variables, both physiologically (e.g., interventricular dependence, pericardial compliance, left atrial compliance, respiratory swings, and intrathoracic pressures) and artificially (zeroing the transducer, overwedging, and incomplete occlusion) [13,16]. Lowering the PAWP cut-off to 12 mmHg has been proposed [17], but a secondary analysis of our data based on PAWP ≤ 12 versus >12 mmHg did not result in better between-group discrimination than the 15 mmHg cut-off, and the proportion of patients who were reclassified when applying the HFpEF criteria was comparable.

Using PAWP as the sole criterion to distinguish between pre- and post-capillary PH in patients with left heart disease has been challenged by others as well, and it has been proposed to interpret PAWP in the clinical context [18,19]. This is illustrated by our study, which identified a cohort of patients with normal left-sided filling pressures despite echocardiographic indices indicating advanced HFpEF [20]. In patients with HFpEF, PAWP can be normal at rest, with intermittent rises during exercise, hypertensive episodes, or fluid retention [21,22,23]. Those intermittent peaks may initiate the development of pulmonary vasculopathy and PH [21,23,24,25]. Hence, a normal PAWP at rest does not exclude HFpEF causing PH. Provocative maneuvers such as exercise or volume challenge during right heart catheterization to uncover HFpEF in patients with PAWP ≤ 15 mmHg at rest have been proposed [21,26], but such tools are invasive, lack reliable cut-off values, and are not considered the standard of care [1]. It may be more straightforward and reasonable to rely on established non-invasive HFpEF criteria rather than non-validated invasive measurements to diagnose PH-HFpEF. However, the diagnostic criteria for HFpEF may require refinement in patients with coexisting PH, the presence of which impacts both the H2FPEF and the HFA-PEFF score—two widely used HFpEF scoring systems [9,11,27]. The diagnostic accuracy of these scores in patients with PH has been questioned before [28,29], and the use of these scores has not been endorsed by recent guidelines [30,31,32].

Abandoning the PAWP criterion for distinguishing between IPAH and PH-HFpEF would have significant consequences. Our data suggest that a considerable proportion of patients currently classified as IPAH, with risk factors for left heart disease, would be re-classified as PH-HFpEF—primarily elderly individuals with cardiometabolic co-morbidities. There is ongoing debate about whether and to what extent these patients benefit from PAH medications, as the available evidence is equivocal [8,33,34,35,36]. Such patients, who are often included, albeit underrepresented, in clinical trials of PAH medications, could benefit from a focused study, avoiding the confounding effects of patients with true IPAH. At the same time, therapeutic interventions that have recently been shown to be efficacious in patients with HFpEF are of uncertain therapeutic value in patients with PAH [37,38,39], and the same will be true for therapeutic interventions currently under development for patients with PH-HFpEF (e.g., AZD3427, NCT05737940).

Re-classifying patients with PH, HFpEF, and a PAWP ≤ 15 mmHg as PH-HFpEF has restored IPAH to its original characterization, mainly affecting younger individuals with fewer risk factors for other cardiac conditions. For these patients, there is unequivocal evidence from randomized clinical trials showing the beneficial effects of parenteral prostacyclin analogues [40], initial phosphodiesterase-5 inhibitor/endothelin receptor antagonist combination therapy [41,42], and emerging agents such as the activin signaling inhibitor sotatercept [43,44], all of which have uncertain safety and efficacy in elderly patients hitherto classified as IPAH with co-morbidities.

Our study has limitations, including its retrospective, single-center setting and a relatively small number of patients due to the rigorous selection criteria. The use of non-standardized HFpEF criteria is an important caveat, owing to the absence of validated HFpEF criteria in patients with an established PH diagnosis. We did not perform exercise or volume challenge during right heart catheterization or other provocative maneuvers such as cardiopulmonary exercise testing with or without echocardiography, which would have provided further information to classify the patients in our study. We included patients with PH-HFpEF and PAWP > 15 mmHg who received treatment with approved PAH medications, acknowledging that such treatment is controversial and not supported by current guidelines [1]. Finally, our study did not include patients with other forms of PAH, such as those with connective tissue disease, in whom PAH and HFpEF may co-exist.

In summary, the current PH classification based on a PAWP threshold of ≤15 mmHg or >15 mmHg may have limitations, especially in patients with HFpEF. Our study proposes a shift from the traditional reliance on PAWP to incorporating HFpEF criteria for PH classification, resulting in a significant re-classification of patients from IPAH to PH-HFpEF and a clearer discrimination between disease phenotypes. Notably, patients with PH-HFpEF and a PAWP ≤ 15 mmHg exhibit similar disease characteristics to those with PAWP > 15 mmHg but differ from IPAH patients. If corroborated by others, these findings could prompt a refined PH classification system that considers not only hemodynamic parameters but also clinical disease phenotypes.

## Figures and Tables

**Table 1:** Original HFA-PEFF score and parameters used in the present study (in red).

	Functional	Morphological	Biomarker (Sinus Rhythm)	Biomarker (Atrial Fibrillation)
Major criteria (2 points)	Septal e′ < 7 cm/s or lateral e′ < 10 cm/s or average E/e′ ≥ 15 or TR velocity > 2.8 m/s	LAVI > 34 mL/m^2^ or LVMI ≥ 149/122 g/m^2^ (m/w) and RWT > 0.42	NT-proBNP > 220 pg/mL or BNP > 80 pg/mL	NT-proBNP > 660 pg/mL or BNP > 240 pg/mL
Minor criteria (1 point)	Average E/e′ 9–14 or GLS < 16%	LAVI 29–34 mL/m^2^ or LVMI ≥ 115/95 g/m^2^ (m/w) or RWT > 0.42 or LV wall thickness ≥ 12 mm	NT-proBNP 125–220 pg/mL or BNP 35–80 pg/mL	NT-proBNP 365–660 pg/mL or BNP 105–240 pg/mL

Definition of abbreviations: e′, mitral annular early diastolic velocity; E/e′, left ventricular filling pressure estimate; GLS, global longitudinal strain; LAVI, left atrial volume index; LVMI, left ventricular mass index; LV, left ventricle; RWT, relative wall thickness; BNP, brain natriuretic peptide.

**Table 2:** Patient characteristics at time of diagnosis.

	IPAH (i) n = 105	*p*-Value (i) vs. (ii)	PH-HFpEF with PAWP ≤ 15 mmHg (ii) n = 115	*p*-Value (ii) vs. (iii)	PH-HFpEF with PAWP > 15 mmHg (iii) n = 130
Age, years	41 [33, 51]	<0.001	76 [69, 80]	0.022	73 [69, 77]
Female	76 (72%)	0.75	81 (70%)	0.332	84 (65%)
BMI, kg/m^2^	26 [23, 30]	0.07	28 [25, 34]	0.108	30 [26, 35]
WHO-FC		0.045		0.432	
I	4 (4%)		0 (0%)		3 (2%)
II	17 (16%)		11 (10%)		12 (9%)
III	79 (75%)		101 (88%)		111 (85%)
IV	5 (5%)		3 (3%)		4 (3%)
6MWD, m	407 [309, 475]	<0.001	278 [213, 372]	0.328	278 [198, 359]
NT-proBNP, ng/L	1049 [260, 2345]	0.204	1142 [516, 2523]	0.459	1694 [664, 3385]
Pulmonary function					
TLC, % pred	101 [90, 110]	<0.001	91 [82, 102]	0.591	89 [78, 98]
FVC, % pred	94 [82, 104]	<0.001	87 [74, 101]	0.784	79 [69, 91]
FEV_1_, % pred	86 [77, 103]	0.003	81 [67, 98]	0.509	74 [62, 86]
FEV_1_/FVC (%)	80 [76, 85]	0.008	77 [71, 81]	0.617	76 [71, 81]
DLCO, % pred	69 [57, 81]	0.384	63 [54, 80]	0.066	59 [47, 77]
PaO_2_, mmHg	73 [65, 80]	<0.001	67 [60–76]	0.685	67 [58, 75]
PaCO_2_, mmHg	33 [30, 35]	<0.001	38 [34, 41]	0.324	39 [35, 42]
Smoking status					
Never	41 (39%)	<0.001	73 (64%)	0.09	60 (46%)
Former	44 (42%)		39 (34%)		66 (51%)
Active	20 (19%)		3 (3%)		4 (3%)
Pack years	11 [4, 20]	0.033	20 [9, 30]	0.497	20 [7, 39]
Comorbidities					
BMI > 30 kg/m^2^	19 (18%)	0.088	32 (28%)	0.101	49 (38%)
Hypertension	36 (34%)	<0.001	97 (84%)	0.669	107 (82%)
CAD	3 (3%)	0.555	5 (4%)	0.386	9 (7%)
Diabetes mell	6 (6%)	<0.001	45 (39%)	0.631	47 (36%)
Atrial fibrillation	2 (2%)	<0.001	76 (66%)	0.809	84 (64%)
Hemodynamics					
RAP, mmHg	8 [4, 12]	0.227	6 [4, 10]	<0.001	12 [9, 15]
mPAP, mmHg	50 [44, 58]	<0.001	32 [27, 40]	<0.001	43 [36, 50]
PAWP, mmHg	8 [6, 11]	<0.001	11 [9, 14]	<0.001	20 [17, 23]
CI, L/min/m^2^	1.8 [1.5, 2.5]	0.44	2.4 [1.9, 2.7]	0.688	2.1 [1.9, 2.6]
PVR, WU	11.1 [8.5, 16.5]	<0.001	4.8 [3.6, 6.7]	0.306	5.3 [3.8, 7.0]
SvO_2_, %	61 [54, 67]	0.002	68 [61, 71]	0.001	63 [58, 68]
Risk (4-strata model) ^a^		0.096		0.019	
Low	14 (14%)		6 (5%)		2 (2%)
Intermediate-low	32 (31%)		29 (25%)		22 (17%)
Intermediate-high	48 (46%)		67 (58%)		72 (57%)
High	10 (10%)		13 (11%)		31 (24%)
Diuretics use at baseline	63 (60%)	0.002	91 (79%)	0.341	109 (84%)
Initial PH medication ^b^					
CCB	14 (18%)	<0.001	0 (0%)	-	0 (0%)
ERA	70 (67%)	<0.001	5 (4%)	0.37	3 (2%)
PDE5i	91 (87%)	<0.001	114 (99%)	0.287	130 (100%)
sGCs	10 (10%)	0.003	1 (1%)	0 (0%)	0.287
PPA	21 (20%)	<0.001	0 (0%)	0 (0%)	-
Monotherapy	29 (28%)		110 (96%)		127 (98%)
Dual combination therapy	53 (51%)		5 (4%)		3 (2%)
Triple combination therapy	23 (22%)	<0.001	0 (0%)	0.37	0 (0%)
PH medication at 1 year					
CCB	11 (11%)	<0.001	0 (0%)	-	0 (0%)
ERA	88 (84%)	<0.001	9 (8%)	0.098	4 (3%)
PDE5i	83 (79%)	0.502	95 (83%)	0.828	106 (82%)
sGCs	16 (15%)	<0.001	1 (1%)	0.469 *	0 (0%)
PPA	39 (37%)	<0.001	2 (2%)	0.219 *	0 (0%)
Monotherapy	10 (10%)		87 (76%)		102 (79%)
Dual combination therapy	53 (51%)		7 (6%)		4 (3%)
Triple combination therapy	40 (38%)	<0.001	2 (2%)	0.297	0 (0%)

Categorical data are shown as n and (%) of the respective population. Continuous data are depicted as median [Q1, Q3]. * Fisher’s Exact Test. ^a^ Risk was determined by the COMPERA 2.0 model [14]. ^b^ Initiated within 3 months after PH diagnosis. Definition of abbreviations: BMI, body mass index; IPAH, idiopathic pulmonary arterial hypertension; PH, pulmonary hypertension; HFpEF, heart failure with preserved ejection fraction; CAD, coronary artery disease; WHO-FC, World Health Organization Functional Class; 6MWD, 6 min walk distance; NT-proBNP, N-terminal fragment of pro-brain natriuretic peptide; TLC, total lung capacity; FVC, forced vital capacity; FEV_1_, forced expiratory volume in 1 s; DLCO, diffusion capacity of the lung for carbon monoxide; RAP, right atrial pressure; mPAP, mean pulmonary arterial pressure; PAWP, pulmonary arterial wedge pressure; CI, cardiac index; PVR, pulmonary vascular resistance; SvO_2_, mixed-venous oxygen saturation; CCB, calcium channel blocker; ERA, endothelin receptor antagonists; PDE5i, phosphodiesterase-5 inhibitors; sGCs; soluble guanylate cyclase stimulator; PPA, prostacyclin pathway agents.

**Table 3:** Echocardiographic parameters at time of diagnosis.

	IPAH (i) n = 105	*p*-Value (i) vs. (ii)	PH-HFpEF with PAWP ≤ 15 mmHg (ii) n = 115	*p*-Value (ii) vs. (iii)	PH-HFpEF with PAWP > 15 mmHg (iii) n = 130
TR velocity (m/s)	3.7 [3.1, 4.2]	0.007	3.4 [3.0, 3.8]	0.044	3.5 [3.1, 3.9]
sPAP (mmHg)	63 [48, 84]	0.003	55 [44, 66]	0.034	60 [49, 75]
RAA (cm^2^)	22 [17, 28]	0.359	24 [19, 30]	0.003	27 [23, 32]
TAPSE (mm)	20 [17, 24]	0.272	18 [15, 23]	0.117	18 [15, 20]
TAPSE/sPAP (mm/mmHg)	0.31 [0.20, 0.49]	0.676	0.34 [0.22, 0.50]	0.004	0.30 [0.21, 0.4]
e′ lateral (cm/s)	10 [7.3, 12.7]	0.031	7.3 [5.8, 9.0]	0.804	7.8 [5.7, 9.3]
e′ septal (cm/s)	5.8 [4.2, 7.8]	0.265	5.0 [4.0, 6.2]	0.478	5.1 [3.9, 6.5]
E/e′	7.6 [5.8, 9.4]	<0.001	13.1 [10.3, 16.5]	0.073	15.2 [10.9, 21.0]
LAVI (mL/m^2^)	19 [12, 25]	<0.001	45 [34, 62]	0.898	48 [37, 60]
LVMI (g/m^2^)	72 [57, 81]	0.017	101 [81, 116]	0.017	108 [86, 129]
LV posterior wall thickness (mm)	9 [8, 11]	<0.001 *	11 [9, 12]	0.038 *	12 [10, 13]
Interventricular wall thickness (mm)	10 [9, 12]	<0.001 *	11 [10, 12]	0.028 *	12 [10, 13]
RV/LV diameter ratio	1.13 [0.94, 1.45]	<0.001	0.93 [0.81, 1.12]	0.443	0.98 [0.84, 1.13]
RWT	0.43 [0.38, 0.54]	0.914	0.45 [0.39, 0.54]	0.297	0.46 [0.40, 0.54]

Categorical data are shown as n and (%) of the respective population. Continuous data are depicted as median [Q1, Q3]. * Non-parametric Wilcoxon rank sum tests due to non-normal distribution of the data. Definition of abbreviations: IPAH, idiopathic pulmonary arterial hypertension; PH, pulmonary hypertension; HFpEF, heart failure with preserved ejection fraction; TR, tricuspid regurgitation; sPAP, systolic pulmonary arterial pressure; RAA, right atrial area; TAPSE, tricuspid annular plane systolic excursion; e′, mitral annular early diastolic velocity; E/e′, left ventricular filling pressure estimate; LAVI, left atrial volume index; LVMI, left ventricular mass index; RV, right ventricle; LV, left ventricle; RWT, relative wall thickness.

## Data Availability

The data presented are available from the corresponding author upon reasonable request.

## Supplementary Materials

The following supporting information can be downloaded at: https://www.mdpi.com/article/10.3390/jcm13247582/s1. Table S1. Patient characteristics at time of diagnosis when grouped by PAWP ≤ 15 mmHg versus PAWP > 15 mmHg. Table S2. Baseline characteristics of patients with PAWP > 15 mmHg who had no signs of HFpEF and were classified as IPAH. Table S3. Echocardiographic parameters in PH-HFpEF patients grouped by without atrial fibrillation versus atrial fibrillation. Table S4. Echocardiographic parameters in patients grouped by PAWP ≤ 15 mmHg versus >15 mmHg. Table S5. Patient characteristics at time of diagnosis when grouped by PAWP ≤ 12 mmHg versus PAWP > 12 mmHg. Table S6. Echocardiographic parameters in patients grouped by PAWP ≤ 12 mmHg versus >12 mmHg. Figure S1. Age distribution of patients grouped by PAWP ≤ 15 mmHg versus >15 mmHg. Figure S2. Baseline and first follow-up measurement for (A) WHO functional class (FC), (B) 6 min walk distance (6MWD), (C) N-terminal fragment of pro-brain natriuretic peptide (NT-proBNP) and (D) mortality risk (as determined by the ESC/ERS 4-strata model) in patients grouped by PAWP ≤ 15 mmHg versus >15 mmHg. Figure S3: Kaplan–Meier survival estimates (A) and age-adjusted survival estimates (B) for patients grouped by PAWP ≤ 15 mmHg versus >15 mmHg. Figure S4: Flow chart when grouped by PAWP ≤ 12 mmHg versus >12 mmHg. Figure S5: Age distribution of patients grouped by PAWP ≤ 12 mmHg versus >12 mmHg. Figure S6: Baseline and first follow-up measurement for (a) functional class (FC), (b) 6 min walk distance (6MWD), (c) N-terminal fragment of pro-brain natriuretic peptide (NT-proBNP), and (d) mortality risk (as determined by the ESC/ERS 4-strata model) in patients grouped by PAWP ≤ 12 mmHg versus >12 mmHg. Figure S7: Kaplan–Meier survival estimates (A) and age-adjusted survival estimates (B) for patients grouped by PAWP ≤ 12 mmHg versus >12 mmHg.
