# Supplementary material for: Reassessing Pulmonary Hypertension Classification: Utilizing Criteria for Heart Failure with Preserved Ejection Fraction Instead of Pulmonary Arterial Wedge Pressure
Source: J Clin Med. 2024 Dec 13;13(24):7582. doi: 10.3390/jcm13247582 (PMC11728240; doi:10.3390/jcm13247582)
Supplement: Supplementary file 1 [file jcm-13-07582-s001.zip › jcm-3333029-supplementary.pdf]

Reassessing Pulmonary Hypertension Classification: Utilizing Criteria for Heart Failure with Preserved Ejection Fraction instead of Pulmonary Arterial Wedge Pressure

Park, DH et al.

Supplemental Material

Table of content:

Table S1. Patient characteristics at time of diagnosis when grouped by PAWP ≤15 mmHg versus PAWP >15 mmHg.

Table S2. Baseline characteristics of patients with PAWP >15 mmHg who had no signs of HFpEF and were classified as IPAH.

Table S3: Echocardiographic parameters in PH-HFpEF patients grouped by without atrial fibrillation versus with atrial fibrillation.

Table S4. Echocardiographic parameters in patients grouped by PAWP ≤15 mmHg versus >15 mmHg.

Table S5. Patient characteristics at time of diagnosis when grouped by PAWP ≤12 mmHg versus PAWP >12 mmHg.

Table S6. Echocardiographic parameters in patients grouped by PAWP ≤12 mmHg versus >12 mmHg.

Figure S1. Age distribution of patients grouped by PAWP ≤15 mmHg versus >15 mmHg.

Figure S2. Baseline and first follow-up measurement for (A) functional class (FC), (B) 6-minute walk distance (6MWD), (C) N-terminal fragment of pro-brain natriuretic peptide (NT-proBNP) and (D) mortality risk (as determined by the ESC/ERS 4-strata model) in patients grouped by PAWP ≤15 mmHg versus >15 mmHg.

Figure S3: Kaplan-Meier survival estimates (A) and age-adjusted survival estimates (B) for patients grouped by PAWP ≤15 mmHg versus >15 mmHg.

Figure S4: Flow chart when grouped by PAWP ≤12 mmHg versus >12 mmHg.

Figure S5: Age distribution of patients grouped by PAWP ≤12 mmHg versus >12 mmHg.

Figure S6: Baseline and first follow-up measurement for (A) functional class (FC), (B) 6-minute walk distance (6MWD), (C) N-terminal fragment of pro-brain natriuretic peptide (NT-proBNP) and (D) mortality risk (as determined by the ESC/ERS 4-strata model) in patients grouped by PAWP ≤12 mmHg versus >12 mmHg.

Figure S7: Kaplan-Meier survival estimates (A) and age-adjusted survival estimates (B) for patients grouped by PAWP ≤12 mmHg versus >12 mmHg.

Table S1. Patient characteristics at time of diagnosis when grouped by PAWP ≤15 mmHg versus PAWP >15 mmHg.

	All patients (n=350)	PAWP ≤ 15 mmHg (n=214)	PAWP >15 mmHg (n=136)	P-value
Age, years	70 [52, 77]	65 [44, 76]	72 [68, 77]	<0.001
Female	241 (69%)	153 (72%)	88 (65%)	0.181
BMI, kg/m^2	28 [25, 34]	27 [24, 33]	30 [25, 35]	0.001
WHO-FC				
I	7 (2%)	4 (2%)	3 (2%)	0.844
II	40 (11%)	27 (13%)	13 (10%)	
III	291 (83%)	176 (83%)	115 (85%)	
IV	12 (3%)	7 (3%)	5 (4%)	
6MWD, m	310 [225, 405]	340 [248, 428]	278 [195, 361]	<0.001
NT-proBNP, ng/L	1248 [493, 2701]	1086 [399, 2427]	1694 [664, 3385]	0.116
Pulmonary function				
TLC, % pred	90 [81, 103]	92 [83, 105]	88 [76, 98]	0.937
FVC, % pred	81 [68, 95]	86 [72, 100]	75 [61, 89]	0.297
FEV_1, % pred	78 [62, 91]	82 [66, 97]	72 [57, 84]	0.107
FEV_1/FVC (%)	78 [72, 82]	78 [72, 84]	76 [71, 81]	0.051
DLCO, % pred	63 [52, 78]	66 [54, 80]	59 [47, 75]	0.011
PaO_2, mmHg	79 [61, 77]	70 [61, 78]	68 [61, 76]	0.525
$PaCO_2$, mmHg	36 [32, 40]	35 [31, 39]	38 [33, 42]	0.003
Smoking status				
Never	174 (49%)	111 (52%)	63 (46%)	0.017
Former	152 (43%)	81 (38%)	68 (50%)	
Active	27 (8%)	22 (10%)	5 (4%)	
Pack years	15 [5, 30]	14 [5, 23]	20 [7, 38]	0.029
Comorbidities				
BMI >30 kg/m^2	100 (29%)	49 (23%)	51 (38%)	0.003
Hypertension	240 (69%)	130 (61%)	110 (81%)	<0.001
CAD	17 (5%)	8 (4%)	9 (7%)	0.222
Diabetes mell.	98 (28%)	51 (24%)	47 (35%)	0.029
Atrial fibrillation	162 (46%)	78 (36%)	84 (62%)	<0.001
Haemodynamics				
RAP, mmHg	10 [6, 13]	7 [4, 11]	12 [9, 15]	<0.001

mPAP, mmHg	43 [34, 52]	42 [32, 52]	43 [37,51]	0.407
PAWP, mmHg	13 [9, 18]	10 [7, 13]	20 [17, 23]	<0.001
CI, L/min/m^2	2.2 [1.8, 2.6]	2.3 [1.9, 2.6]	2.1 [1.9, 2.6]	0.162
PVR, WU	6.3 [4.2, 9.7]	7.6 [4.6, 11.1]	5.2 [3.8, 7.0]	<0.001
SvO_2, %	64 [58, 70]	66 [58, 71]	63 [58, 68]	0.028
Risk (4-strata model)[a]				
Low	22 (6%)	19 (9%)	3 (2%)	<0.001
Intermediate-low	83 (24%)	60 (28%)	23 (17%)	
Intermediate-high	187 (54%)	113 (53%)	74 (56%)	
High	54 (16%)	21 (10%)	33 (25%)	
Diuretics use at baseline	263 (75%)	150 (70%)	113 (83%)	0.006
Initial PH medication[b]				
CCB	14 (6%)	14 (7%)	0 (0%)	0.002
ERA	78 (22%)	71 (33%)	7 (5%)	<0.001
PDE5i	335 (96%)	199 (93%)	136 (100%)	0.002
sGCs	11 (3%)	11 (5%)	0 (0%)	0.004*
PPA	21 (6%)	19 (9%)	2 (2%)	0.004
Monotherapy	266 (76%)	137 (64%)	129 (95%)	<0.001
Dual combination therapy	61 (17%)	56 (26%)	5 (4%)	
Triple combination therapy	23 (7%)	21 (10%)	2 (2%)	
PH medication at				
1 year	11 (3%)	11 (5%)	0 (0%)	0.004*
CCB	100 (29%)	91 (43%)	10 (7%)	<0.001
ERA	282 (61%)	173 (81%)	111 (82%)	0.856
PDE5i	19 (5%)	16 (8%)	1 (1%)	0.004
sGCs	40 (11%)	37 (17%)	4 (3%)	<0.001
PPA				
Monotherapy	200 (57%)	97 (45%)	102 (75%)	<0.001
Dual combination therapy	64 (18%)	58 (27%)	6 (4%)	
Triple combination therapy	41 (12%)	38 (18%)	4 (3%)	

Categorical data are shown as n and (%) of the respective population. Continuous data are depicted as median [Q1, Q3]. *Fishers Exact Test

[a]Risk was determined by the COMPERA 2.0 model [1].

[b]initiated within 3 months after PH diagnosis.

Definition of abbreviations: BMI, body mass index; IPAH, idiopathic pulmonary arterial hypertension; PH, pulmonary hypertension; HFpEF, heart failure with preserved ejection fraction; CAD, coronary artery disease; WHO-FC, World Health Organization Functional Class; 6MWD, 6-minute walk distance; NT-proBNP, N-terminal fragment of pro-brain natriuretic peptide; TLC, total lung capacity; FVC, forced vital capacity; FEV_1, forced expiratory volume in 1 s; DLCO, diffusion capacity of the lung for carbon monoxide; RAP, right atrial pressure; mPAP, mean pulmonary arterial pressure; PAWP, pulmonary arterial wedge pressure; CI, cardiac index; PVR, pulmonary vascular resistance; SvO_2, mixed-venous oxygen saturation; CCB, calcium channel blocker; ERA, endothelin receptor antagonists; PDE5i, phosphodiesterase-5 inhibitors; sGCs, soluble guanylate cyclase stimulator; PPA, prostacyclin pathway agents.

Table S2. Baseline characteristics of patients with PAWP >15 mmHg who had no signs of HFpEF and were classified as IPAH.

Pat ID	Age (yrs)	Sex (m/ f)	BMI (kg/ m²)	HTN (y/n)	DM (y/n)	CAD (y/n)	RAP (mmHg)	mPAP (mmHg)	PAWP (mmHg)	PVR (WU)	E/e’	LAVI (ml/ m²)	LVMI (g/m²)
1	45	f	22.3	n	n	n	10	47	21	9,6	5.8	14.2	92.9
2	28	m	26.9	n	n	n	2	43	21	4,2	4	17.1	60.4
3	47	m	21.9	y	n	n	3	52	16	10,7	8.7	20	70.3
4	30	f	50.3	y	n	n	29	81	17	20	8.5	16	76.9
5	24	f	32.6	n	n	n	6	42	21	6.3	8.7	13	86.6
6	45	f	38.1	y	n	n	25	55	18	20.9	11.6	23.6	65.9

Definition of abbreviations: f, female; m, male; BMI, body mass index; HTN, arterial hypertension; DM, diabetes mellitus; CAD, coronary heart disease; IPAH, idiopathic pulmonary arterial hypertension; mPAP, mean pulmonary arterial pressure; PVR, pulmonary vascular resistance; PAWP, pulmonary arterial wedge pressure; E/e’, left ventricular filling pressure estimate; LAVI, left atrial volume index; LVMI, left ventricular mass index.

Table S3. Echocardiographic parameters in PH-HFpEF patients grouped by without atrial fibrillation versus with atrial fibrillation

	all PH-HFpEF patients (n=246)	PH-HFpEF without atrial fibrillation (n=85)	PH-HFpEF with atrial fibrillation (n=160)	P-value
TR velocity (m/s)	3.5 [3.1, 3.9]	3.4 [3.2, 3.9]	3.5 [3.0, 3.9]	0.379
sPAP (mmHg)	56 [47, 70]	56 [47,73]	57 [47, 70]	0.704
RAA (cm^2)	25 [21, 31]	24 [19, 31]	26 [22, 32]	0.023
TAPSE (mm)	18 [15, 22]	20 [17, 24]	17 [15, 20]	<0.001
TAPSE/sPAP (mm/mmHg)	0.32 [0.21, 0.43]	0.34 [0.24, 0.47]	0.30 [0.21, 0.40]	0.067
e' lateral (cm/s)	7.6 [5.8, 9.3]	7.0 [5.6, 8.6]	7.8 [5.9, 9.7]	0.024
e' septal (cm/s)	5.1 [3.9, 6.3]	4.7 [3.9, 5.8]	5.3 [4.0, 6.5]	0.009
E/e'	14.1 [10.5, 19.6]	12.7 [9.9, 16.6]	15.2 [10.9, 20.2]	0.336
LAVI (ml/m^2)	46 [34, 60]	40 [29, 53]	49 [39, 62]	0.001
LVMI (g/m^2)	103 [83, 123]	110 [88, 126]	101 [82, 122]	0.276
LV posterior wall thickness (mm)	11 [10, 12]	12 [10, 13]	11 [9, 12]	0.006
Interventricular septum thickness (mm)	11 [10, 13]	12 [10, 13]	11 [10, 13]	0.839
RV/LV diameter ratio	0.96 [0.82, 1.13]	0.98 [0.810, 0.16]	0.96 [0.84, 1.11]	0.347
RWT	0.45 [0.40, 0.54]	0.48 [0.41, 0.56]	0.45 [0.38, 0.53]	0.246

Categorical data are shown as n and (%) of the respective population. Continuous data are depicted as median [Q1, Q3].

Definition of abbreviations: PAWP, pulmonary arterial wedge pressure; TR, tricuspid regurgitation; sPAP, systolic pulmonary arterial pressure; RAA, right atrial area; TAPSE, tricuspid annular plane systolic excursion; e', mitral annular early diastolic velocity; E/e',

left ventricular filling pressure estimate; LAVI, left atrial volume index; LVMI, left ventricular mass index; RV, right ventricle; LV, left ventricle; RWT, relative wall thickness.

Table S4. Echocardiographic parameters in patients grouped by PAWP ≤15 mmHg versus >15 mmHg.

	All patients (n=350)	PAWP ≤ 15 mmHg (n=214)	PAWP >15 mmHg (n=136)	P-value
TR velocity (m/s)	3.5 [3.1, 4.0]	3.5 [3.1, 4.0]	3.5 [3.2, 3.9]	0.484
sPAP (mmHg)	59 [47, 75]	58 [46, 75]	60 [50, 75]	0.340
RAA (cm^2)	25 [19,31]	23 [18, 29]	27 [23, 32]	<0.001
TAPSE (mm)	18 [16, 23]	19 [16, 23]	18 [15, 20]	0.012
TAPSE/sPAP (mm/mmHg)	0.32 [0.21, 0.44]	0.33 [0.21, 0.49]	0.30 [0.20, 0.40]	0.006
e' lateral (cm/s)	8.0 [6.2, 10.7]	8.4 [6.3, 11.3]	7.9 [5.8, 9.5]	0.609
e' septal (cm/s)	5.2 [4.0, 6.6]	5.3 [4.0, 6.6]	5.1 [4.0, 6.6]	0.196
E/e'	12.0 [8.2, 16.4]	10.5 [7.3, 14.3]	15.1 [10.4, 20.5]	<0.001
LAVI (ml/m^2)	38 [23, 54]	30 [20, 47]	47 [34, 58]	<0.001
LVMI (g/m^2)	91 [73, 114]	83 [68, 104]	105 [83, 128]	<0.001
LV posterior wall thickness (mm)	10 [9, 12]	10 [9, 12]	11 [10, 13]	<0.001
Interventricular septum thickness (mm)	11 [10, 13]	11 [9, 12]	12 [10, 13]	<0.001
RV/LV diameter ratio	1.00 [0.85, 1.20]	1.02 [0.88, 1.30]	0.98 [0.84, 1.13]	0.020
RWT	0.45 [0.39, 0.54]	0.44 [0.38, 0.53]	0.46 [0.40, 0.54]	0.169

Categorical data are shown as n and (%) of the respective population. Continuous data are depicted as median [Q1, Q3].

Definition of abbreviations: PAWP, pulmonary arterial wedge pressure; TR, tricuspid regurgitation; sPAP, systolic pulmonary arterial pressure; RAA, right atrial area; TAPSE, tricuspid annular plane systolic excursion; e', mitral annular early diastolic velocity; E/e',

left ventricular filling pressure estimate; LAVI, left atrial volume index; LVMI, left ventricular mass index; RV, right ventricle; LV, left ventricle; RWT, relative wall thickness.

Table S5. Patient characteristics at time of diagnosis when grouped by PAWP ≤12 mmHg versus PAWP >12 mmHg.

	All patients (n=350)	PAWP ≤ 12 mmHg (n=159)	PAWP >12 mmHg (n=191)	P-value
Age, years	70 [52, 77]	58 [38, 73]	73 [68, 78]	<0.001
Female	241 (69%)	116 (73%)	125 (65%)	0.131
BMI, kg/m^2	28 [25, 34]	27 [24, 33]	29 [25, 34]	0.021
WHO-FC				
I	7 (2%)	4 (2%)	3 (2%)	0.809
II	40 (11%)	20 (13%)	20 (10%)	
III	291 (83%)	129 (81%)	162 (85%)	
IV	12 (3%)	6 (4%)	6 (3%)	
6MWD, m	308 [223, 402]	340 [248, 432]	297 [198, 378]	<0.001
NT-proBNP, ng/L	1248 [493, 2701]	1041 [287, 2406]	1593 [294, 2405]	0.168
Pulmonary function				
TLC, % pred	90 [81, 103]	97 [85, 107]	88 [76, 98]	0.545
FVC, % pred	81 [68, 95]	88 [72, 100]	78 [64, 90]	0.231
FEV_1, % pred	78 [62, 91]	84 [66, 97]	73 [60, 87]	0.119
FEV_1/FVC (%)	78 [72, 82]	79 [72, 84]	76 [72, 81]	0.025
DLCO, % pred	63 [52, 78]	65 [52, 79]	61 [50, 77]	0.256
PaO_2, mmHg	70 [61, 77]	71[61, 78]	68 [61, 76]	0.362
$PaCO_2$, mmHg	36 [32, 40]	33 [30, 38]	38 [34, 42]	<0.001
Smoking status				
Never	174 (49%)	79 (50%)	95 (50%)	0.519
Former	152 (43%)	65 (41%)	84 (44%)	
Active	27 (8%)	15 (9%)	12 (6%)	
Pack years	15 [5; 30]	12 [4, 20]	20 [10, 37]	0.005
Comorbidities				
BMI >30 kg/m^2	100 (29%)	40 (25%)	60 (31%)	0.197
Hypertension	240 (69%)	89 (56%)	151 (79%)	<0.001
CHD	17 (5%)	7 (4%)	10 (5%)	0.718
Diabetes mell.	98 (28%)	31 (20%)	67 (35%)	0.001
Atrial fibrillation	162 (46%)	42 (26%)	120 (63%)	<0.001

Haemodynamics				
RAP, mmHg	10 [6, 13]	6 [4, 10]	11 [9, 16]	<0.001
mPAP, mmHg	43 [34, 52]	44 [33, 55]	41 [34, 50]	0.108
PAWP, mmHg	13 [9, 18]	8 [6, 11]	17 [15, 21]	<0.001
CI, L/min/m^2	2.2 [1.8, 2.6]	2,2 [1.7, 2.6]	2.2 [1.9, 2.7]	0.066
PVR, WU	6.3 [4.2, 9.7]	8.5 [5.6, 12.7]	5 [3.7, 7.0]	<0.001
SvO_2, %	64 [58, 70]	65 [57, 70]	65 [59, 69]	0.421
Risk (4-strata model)[a]				0.004
Low	22 (6%)	14 (9%)	8 (4%)	
Intermediate-low	83 (24%)	46 (29%)	37 (20%)	
Intermediate-high	187 (54%)	83 (53%)	104 (55%)	
High	54 (16%)	15 (9%)	39 (21%)	
Diuretics use at baseline	263 (75%)	108 (68%)	155 (81%)	0.004
Initial PH medication[b]				
CCB	14 (6%)	14 (9%)	0 (0%)	<0.001
ERA	78 (22%)	66 (42%)	12 (6%)	<0.001
PDE5i	335 (96%)	144 (91%)	191 (100%)	<0.001
sGCs	11 (3%)	10 (6%)	1 (0.5%)	0.002
PPA	21 (6%)	19 (12%)	2 (1%)	<0.001
Monotherapy	215 (61%)	87 (55%)	179 (73%)	<0.001
Dual combination therapy	61 (17%)	52 (33%)	9 (5%)	
Triple combination therapy	23 (7%)	20 (13%)	3 (2%)	
PH medication at				
1 year	11 (3%)	11 (7%)	0 (0%)	<0.001
CCB	100 (29%)	79 (50%)	22 (12%)	<0.001
ERA	282 (61%)	128 (81%)	156 (82%)	0.780
PDE5i	19 (5%)	15 (9%)	2 (1%)	<0.001
sGCs	40 (11%)	33 (21%)	8 (4%)	<0.001
PPA				
Monotherapy	199 (57%)	63 (40%)	136 (72%)	<0.001
Dual combination therapy	64 (18%)	50 (31%)	14 (7%)	
	42 (12%)	34 (21%)	8 (4%)	

Triple combination therapy				

Categorical data are shown as n and (%) of the respective population. Continuous data are depicted as median [Q1, Q3]. *Fishers Exact Test

[a]Risk was determined by the COMPERA 2.0 model [1].

[b]initiated within 3 months after PH diagnosis.

Definition of abbreviations: BMI, body mass index; IPAH, idiopathic pulmonary arterial hypertension; PH, pulmonary hypertension; HFpEF, heart failure with preserved ejection fraction; CAD, coronary artery disease; WHO-FC, World Health Organization Functional Class; 6MWD, 6-minute walk distance; NT-proBNP, N-terminal fragment of pro-brain natriuretic peptide; TLC, total lung capacity; FVC, forced vital capacity; FEV_1, forced expiratory volume in 1 s; DLCO, diffusion capacity of the lung for carbon monoxide; RAP, right atrial pressure; mPAP, mean pulmonary arterial pressure; PAWP, pulmonary arterial wedge pressure; CI, cardiac index; PVR, pulmonary vascular resistance; SvO_2, mixed-venous oxygen saturation; CCB, calcium channel blocker; ERA, endothelin receptor antagonists; PDE5i, phosphodiesterase-5 inhibitors; sGCs, soluble guanylate cyclase stimulator; PPA, prostacyclin pathway agents.

Table S6. Echocardiographic parameters in patients grouped by PAWP ≤12 mmHg versus >12 mmHg.

	All patients (n=350)	PAWP ≤ 12mmHg (n=214)	PAWP >12mmHg (n=136)	P-value
TR velocity (m/s)	3.5 [3.1, 4.0]	3.6 [3.1, 4.0]	3.4 [3.1, 3.9]	0.239
sPAP (mmHg)	59 [47, 75]	61 [48, 77]	57 [47, 74]	0.421
RAA (cm^2)	25 [19,31]	23 [17, 29]	26 [21, 32]	0.001
TAPSE (mm)	18 [16, 23]	19 [16, 23]	18 [15, 23]	0.417
TAPSE/sPAP (mm/mmHg)	0.32 [0.21, 0.44]	0.32 [0.20, 0.45]	0.32 [0.21, 0.44]	0.885
e' lateral (cm/s)	8.0 [6.2, 10.7]	8.6 [6.4, 11.7]	7.8 [5.8, 9.7]	0.249
e' septal (cm/s)	5.2 [4.0, 6.6]	5.2 [3.9, 6.6]	5.3 [4.0, 6.6]	0.245
E/e'	12.0 [8.2, 16.4]	9.2 [6.8, 13.0]	14.6 [10.5, 19.4]	<0.001
LAVI (ml/m^2)	38 [23, 54]	26 [17, 42]	46 [34, 59]	<0.001
LVMI (g/m^2)	91 [73, 114]	77 [62, 102]	103 [82, 124]	<0.001
LV posterior wall thickness (mm)	10 [9, 12]	10 [9, 11]	11 [10, 12]	<0.001
Interventricular wall thickness (mm)	11 [10, 13]	11 [9, 12]	11 [10, 13]	<0.001
RV/LV diameter ratio	1.00 [0.85, 1.20]	1.10 [0.88, 1.32]	0.96 [0.82, 1.13]	0.003
RWT	0.45 [0.39, 0.54]	0.44 [0.37, 0.53]	0.45 [0.40, 0.54]	0.364

Categorical data are shown as n and (%) of the respective population. Continuous data are depicted as median [Q1, Q3].

Definition of abbreviations: PAWP, pulmonary arterial wedge pressure; TR, tricuspid regurgitation; sPAP, systolic pulmonary arterial pressure; RAA, right atrial area; TAPSE, tricuspid annular plane systolic excursion; e', mitral annular early diastolic velocity; E/e', left ventricular filling pressure estimate; LAVI, left atrial volume index; LVMI, left ventricular mass index; RV, right ventricle; LV, left ventricle; RWT, relative wall thickness.

Figure S1. Age distribution of patients grouped by PAWP ≤15 mmHg versus >15 mmHg.

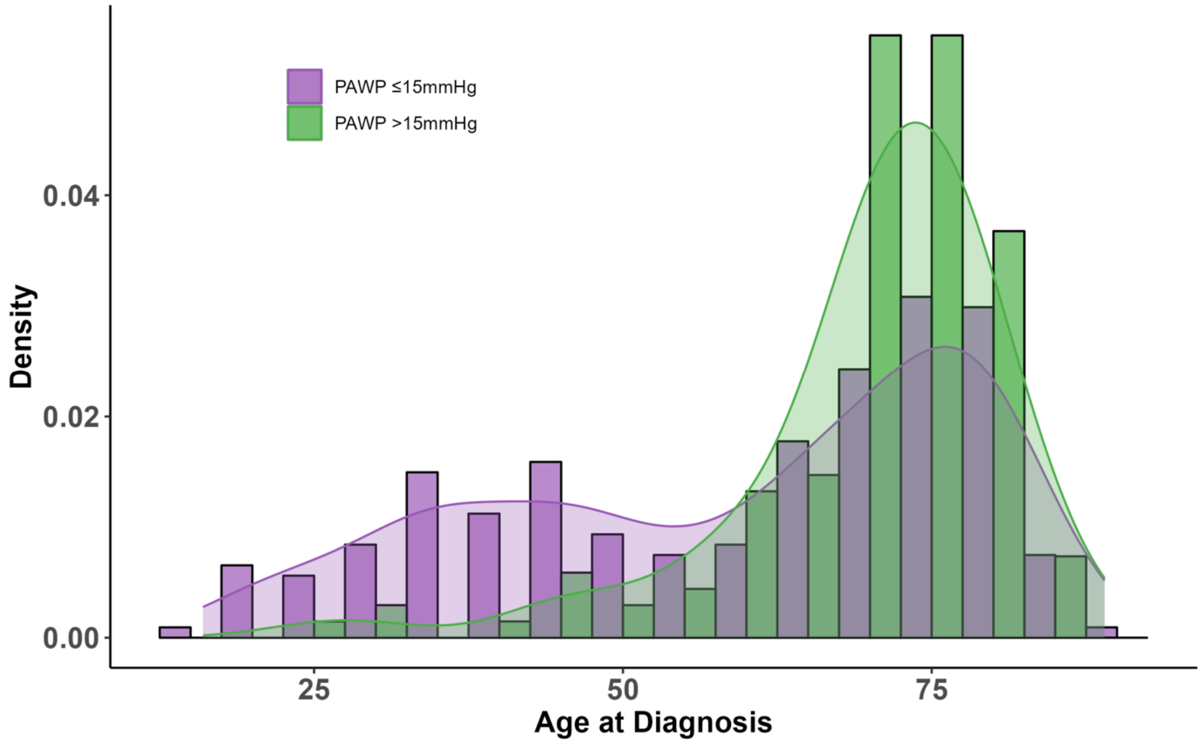

Density plots showing the age distribution of patients with PAWP ≤15 mmHg (purple), and PAWP >15 mmHg (green).

Definition of abbreviations: PAWP, pulmonary arterial wedge pressure.

Figure S2. Baseline and first follow-up measurement for (A) WHO functional class (FC), (B) 6-minute walk distance (6MWD), (C) N-terminal fragment of pro-brain natriuretic peptide (NT-proBNP) and (D) mortality risk (as determined by the ESC/ERS 4-strata model) in patients grouped by PAWP ≤15 mmHg versus >15 mmHg.

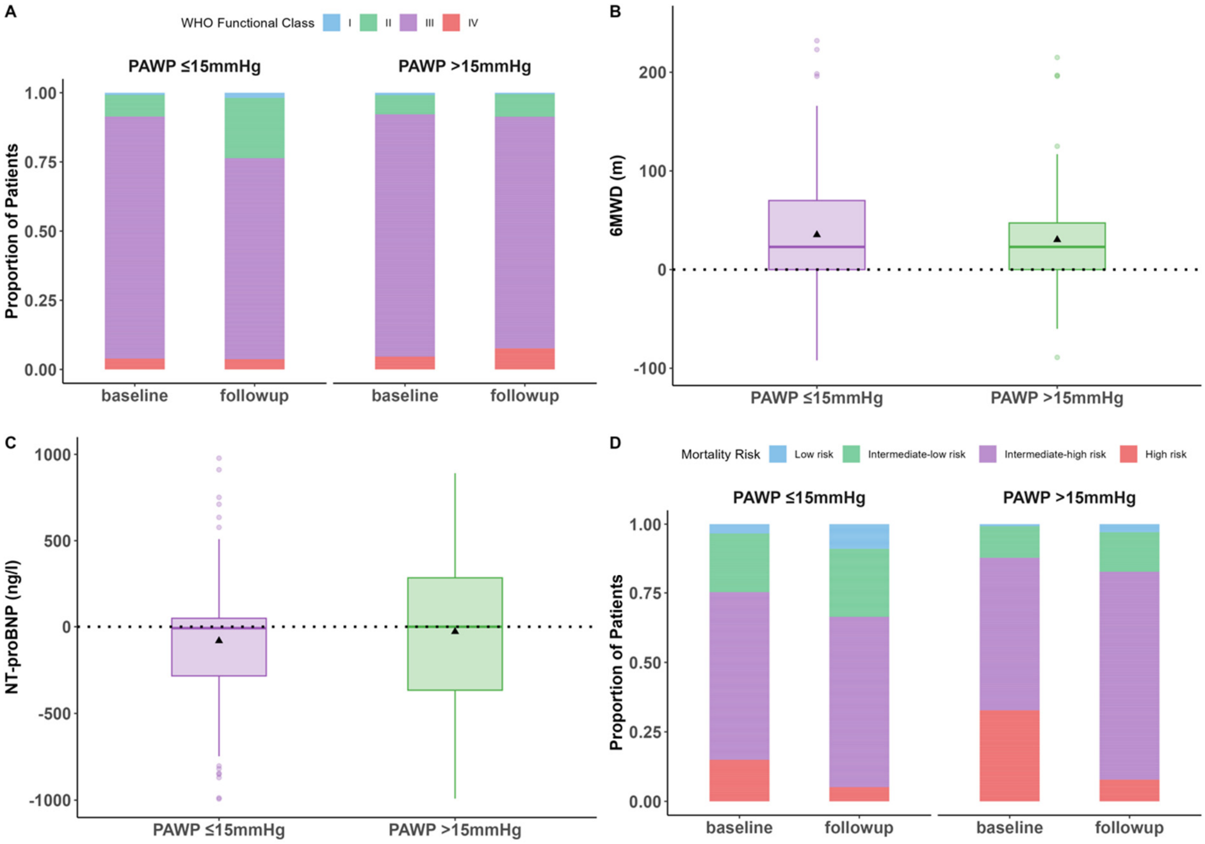

Panel A: Bar graphs of WHO functional class at baseline and first follow-up after treatment initiation. Panel B: Box and whisker plots showing changes from baseline to first follow-up in 6-minute walk distance. Panel C: Box and whisker plots showing changes from baseline to first follow-up in NT-proBNP. Panel D: Bar graphs of mortality risk estimated by the ESC/ERS 4-strata model at baseline and first follow-up.

The triangles in Panels B and C represent the respective mean changes.

Definition of abbreviations: PAWP, pulmonary arterial wedge pressure; 6MWD, 6-minute walk distance; NT-proBNP, N-terminal fragment of pro-brain natriuretic peptide.

Figure S3: Kaplan-Meier survival estimates (A) and age-adjusted survival estimates for patients grouped by PAWP ≤15 mmHg versus >15 mmHg.*

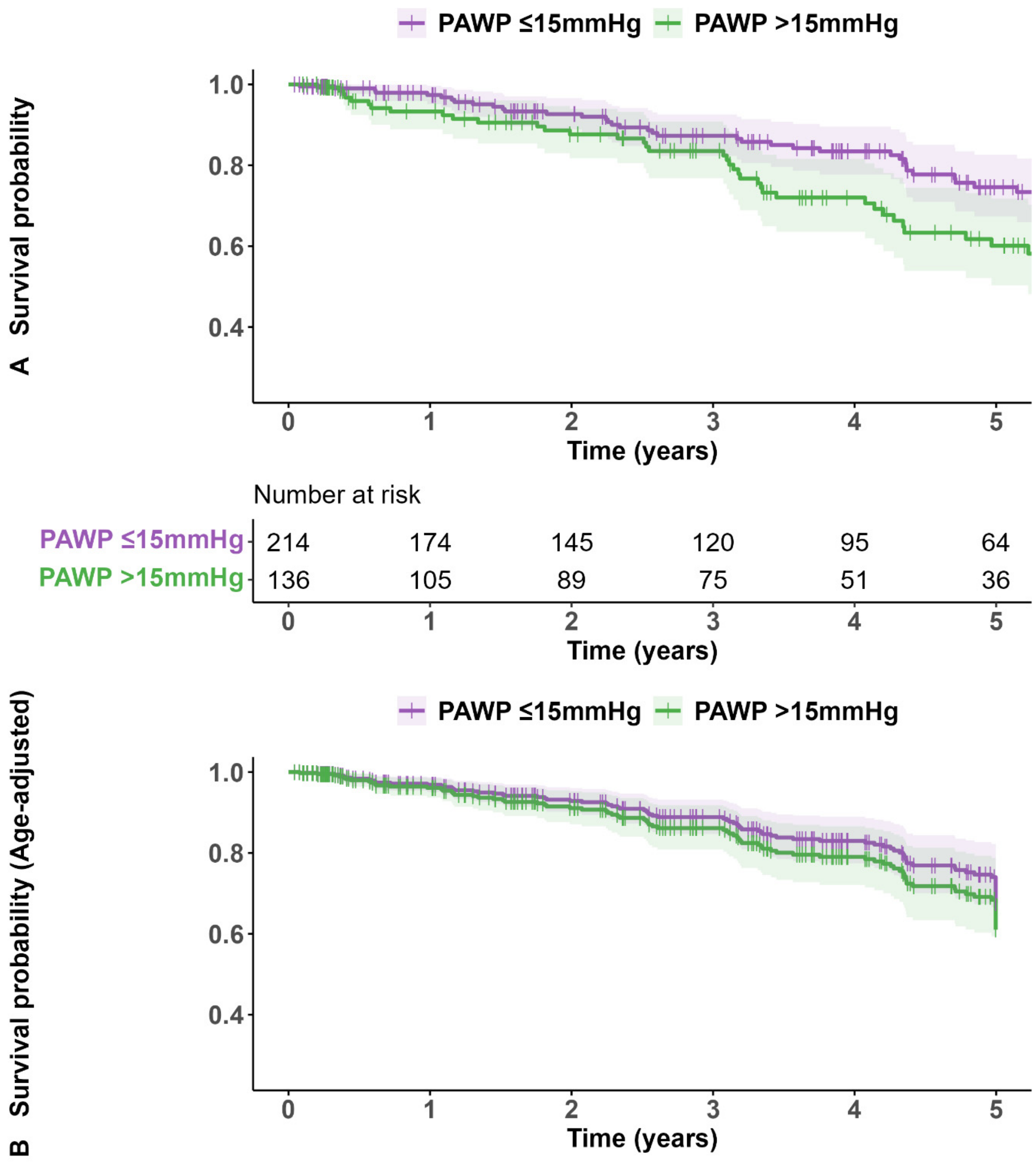

Definition of abbreviations: PAWP, pulmonary arterial wedge pressure. *p=0.017 for the unadjusted analysis and p=0.310 for the age-adjusted analysis.

Figure S4: Flow chart when grouped by PAWP ≤12 mmHg versus >12 mmHg.

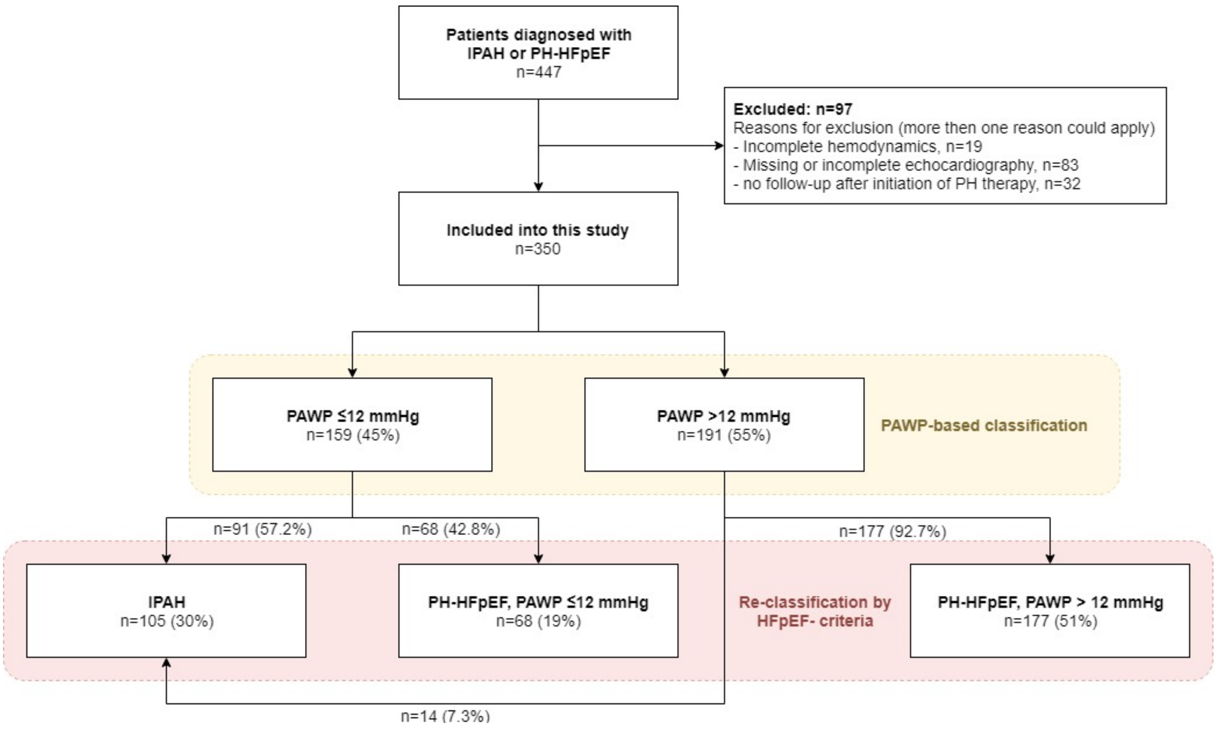

Definition of abbreviations: IPAH, idiopathic pulmonary arterial hypertension; PH, pulmonary hypertension; HFpEF, heart failure with preserved ejection fraction; PAWP, pulmonary arterial wedge pressure.

Figure S5: Age distribution of patients grouped by PAWP ≤12 mmHg versus >12 mmHg.

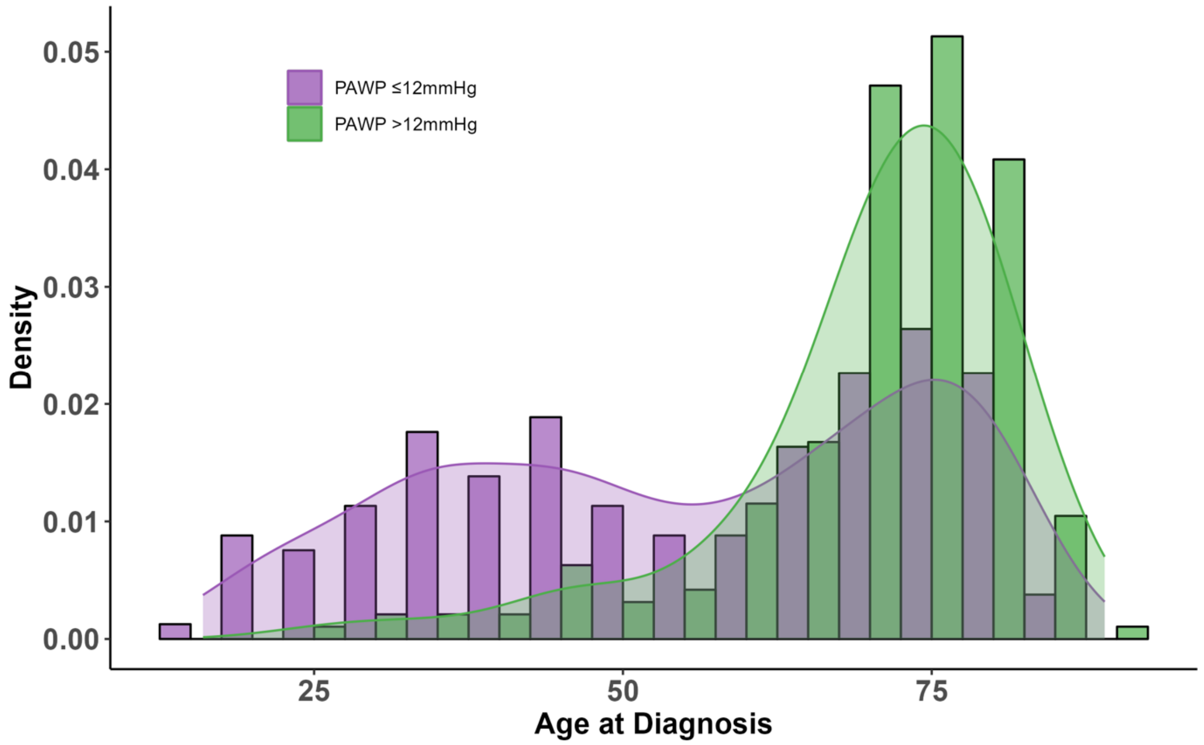

Density plots showing the age distribution of patients with PAWP ≤12 mmHg (purple), and PAWP >12 mmHg (green).

Definition of abbreviations: PAWP, pulmonary arterial wedge pressure.

Figure S6: Baseline and first follow-up measurement for (A) functional class (FC), (B) 6-minute walk distance (6MWD), (C) N-terminal fragment of pro-brain natriuretic peptide (NT-proBNP) and (D) mortality risk (as determined by the ESC/ERS 4-strata model) in patients grouped by PAWP ≤12 mmHg versus >12 mmHg.

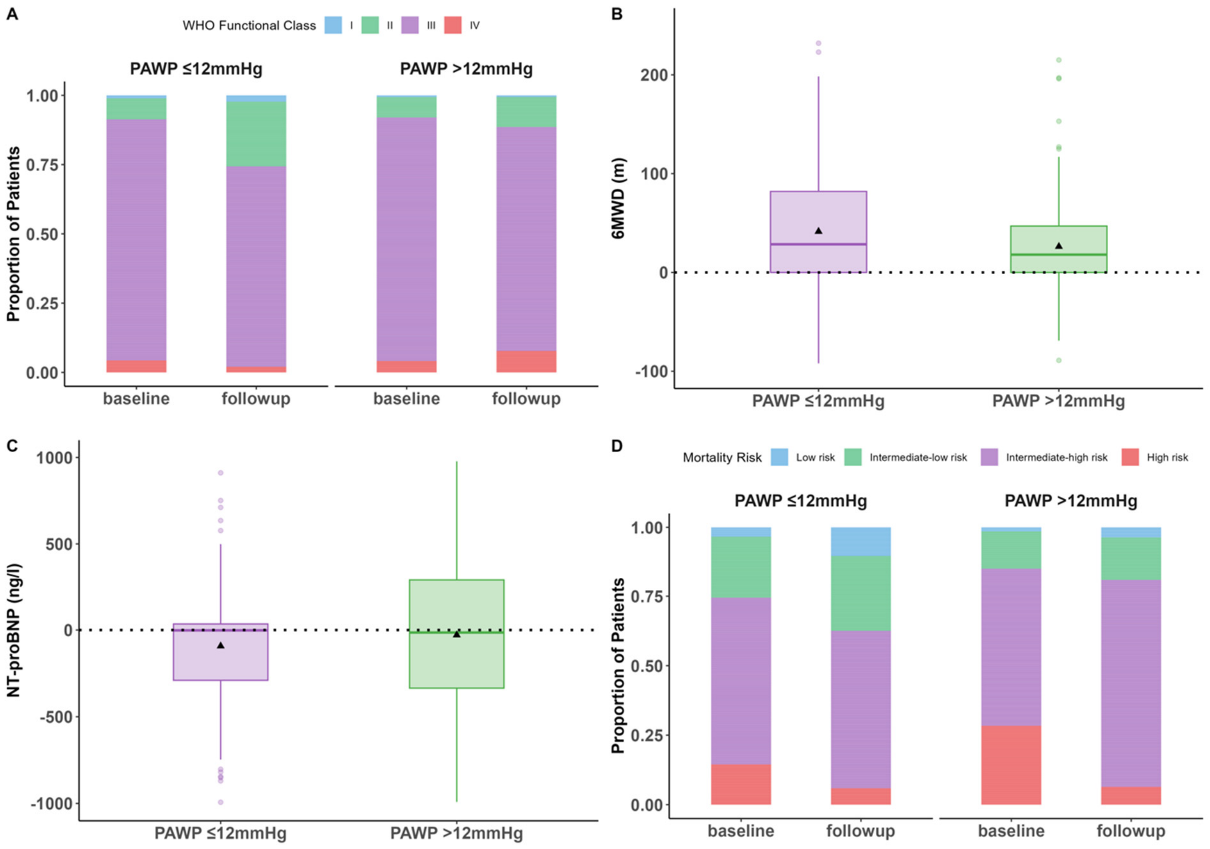

Panel A: Bar graphs of WHO functional class at baseline and first follow-up after treatment initiation. Panel B: Box and whisker plots showing changes from baseline to first follow-up in 6-minute walk distance. Panel C: Box and whisker plots showing changes from baseline to first follow-up in NT-proBNP. Panel D: Bar graphs of mortality risk estimated by the ESC/ERS 4-strata model at baseline and first follow-up.

The triangles in Panels B and C represent the respective mean changes.

Definition of abbreviations: PAWP, pulmonary arterial wedge pressure; 6MWD, 6-minute walk distance; NT-proBNP, N-terminal fragment of pro-brain natriuretic peptide.

Figure S7: Kaplan-Meier survival estimates (A) and age-adjusted survival estimates (B)for patients grouped by PAWP ≤12 mmHg versus >12 mmHg.

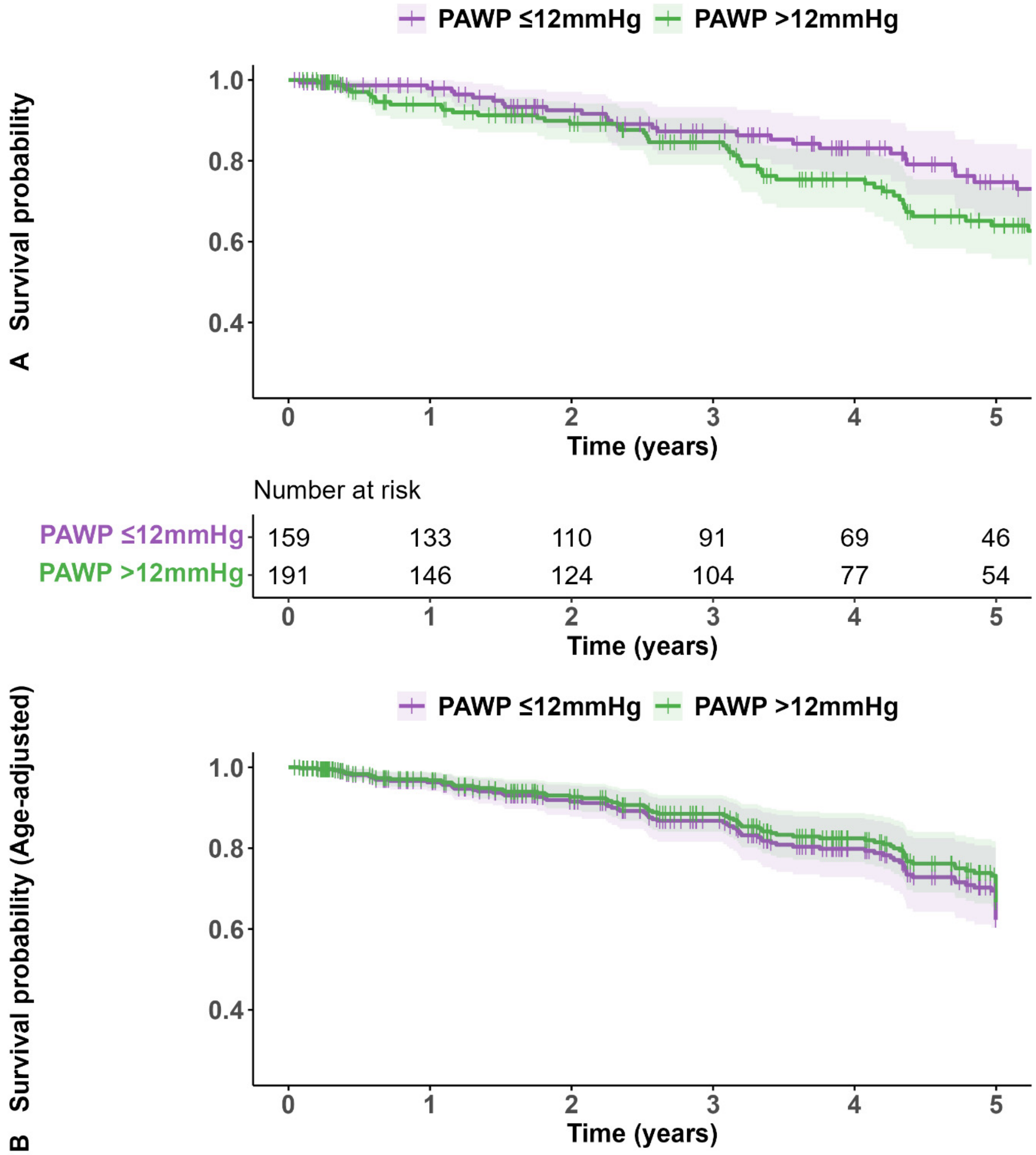

Definition of abbreviations: PAWP, pulmonary arterial wedge pressure. *p=0.073 for the unadjusted analysis and p=0.542 for the age-adjusted analysis

Reference

1. Hoeper MM, Pausch C, Olsson KM, Huscher D, Pittrow D, Grunig E, Staehler G, Vizza CD, Gall H, Distler O, Opitz C, Gibbs JSR, Delcroix M, Ghofrani HA, Park DH, Ewert R, Kaemmerer H, Kabitz HJ, Skowasch D, Behr J, Milger K, Halank M, Wilkens H, Seyfarth HJ, Held M, Dumitrescu D, Tsangaris I, Vonk-Noordegraaf A, Ulrich S, Klose H, Claussen M, Lange TJ, Rosenkranz S. COMPERA 2.0: a refined four-stratum risk assessment model for pulmonary arterial hypertension. *Eur Respir J* 2022: 60(1): 2103311.
